# Use of nanotechnology-based surface antiseptic solutions in the ICU

**DOI:** 10.1186/cc14158

**Published:** 2015-03-16

**Authors:** Y Kuplay, N Akgun, C Agalar, H Aydýn, O Alýcý, G Turan

**Affiliations:** 1 FSM Teaching and Research Hospital, Istanbul, Turkey

## Introduction

In our study, we aimed to compare the application of benzalkonium chloride (BC) - a nanotechnology-based product - for 24-hour periods and didecyl dimethyl ammonium chloride (DDAC) for 12-hour periods regarding efficiency in application of surface antiseptics in the ICU.

## Methods

Two different areas with eight beds at both sides of a common corridor in the ICU were named as areas A and B. BC was applied in area A with 24-hour periods and DDCA was applied in area B with 12hour periods for surface cleaning. Samples were taken from a total of 20 different surfaces including nurse-station desks, phones, keyboards, beds, bedside monitors and ventilators by the same infection control nurse every 24 hours from area A and every 12 hours from area B for 7 days. Swab samples were cultured on 5% sheep bloody agar and McConkey agar in the laboratory. Then the cultured mediums were incubated at 35 to 37°C in an aerobic environment for 18 to 24 hours. NCSS (Number Cruncher Statistical System) 2007 and PASS 2008 Statistical Software (UT, USA) program were used for the statistical analysis.

## Results

There were no statistical differences between two groups (Table 1).

**Table 1:** Isolated pathogen ratio percentage

	A (BC)	B (DDCA)	*P *value
First day	25	20	1.000
Second day	5	15	0.605
Third day	30	20	0.715
Fourth day	65	50	0.527
Fifth day	45	60	0.527
Sixth day	25	25	1.000
Seventh day	60	45	0.527

## Conclusion

The effect of a good surface disinfectant should begin immediately and it should have a long-lasting disinfecting effect on the surface. DDAC is an efficient disinfectant used in medicine and the food industry to protect the surfaces. However, it may cause severe skin itching. BC, which is a nanotechnology-based product, leaves its active metabolites on the surface; it is applied by constituting a spongy layer. Since the efficiency of BC lasts for 24 hours and it is applied to perform cleaning with 24-hour intervals, we think that it is preferable with regards to workforce gain and cost.

